# Oxidative Stress and Lipid Dysregulation in Lipid Droplets: A Connection to Chronic Kidney Disease Revealed in Human Kidney Cells

**DOI:** 10.3390/antiox11071387

**Published:** 2022-07-18

**Authors:** Zhen Chen, Rojeet Shrestha, Xiaoyue Yang, Xunzhi Wu, Jiaping Jia, Hitoshi Chiba, Shu-Ping Hui

**Affiliations:** 1 Faculty of Health Sciences, Hokkaido University, Kita-12, Nishi-5, Kita-ku, Sapporo 060-0812, Japan; chenzhen@hs.hokudai.ac.jp (Z.C.); rshrestha@pclabsdx.com (R.S.); yangxy1349@outlook.com (X.Y.); xunzhi.wu.m6@elms.hokudai.ac.jp (X.W.); jiaping.jia.s1@elms.hokudai.ac.jp (J.J.); 2 Patients Choice Laboratories, Indianapolis, IN 46278, USA; 3 Department of Nutrition, Sapporo University of Health Sciences, Nakanuma Nishi-4-2-1-15, Higashi-ku, Sapporo 007-0894, Japan; chiba-h@sapporo-hokeniryou-u.ac.jp

**Keywords:** lipid droplets (LDs), chronic kidney disease (CKD), oxidative stress, ectopic lipid accumulation, lipid hydroperoxides, triglycerides, phosphatidylcholines, phosphatidylethanolamines, cholesteryl esters, molecular species

## Abstract

Chronic kidney disease (CKD), which is defined as a condition causing the gradual loss of kidney function, shows renal lipid droplet (LD) accumulation that is associated with oxidative damage. There is a possibility that an LD abnormality in quality plays a role in CKD development. This study aimed to explore the chemical composition of LDs that are induced in human kidney cells during exposure to free fatty acids as an LD source and oxidized lipoproteins as oxidative stress. The LDs were aspirated directly from cells using nanotips, followed by in-tip microextraction, and the LD lipidomic profiling was conducted using nanoelectrospray mass spectrometry. As a result, the free fatty acids increased the LD lipid content and, at the same time, changed their composition significantly. The oxidized lipoproteins caused distorted proportions of intact lipids, such as triacylglycerols (TG), phosphatidylcholines (PC), phosphatidylethanolamines (PE), and cholesteryl esters (CE). Notably, the oxidized lipids, including the hydroperoxides of TG, PC, and PE, exhibited significant elevations in dose-dependent manners. Furthermore, the dysregulation of intact lipids was paralleled with the accumulation of lipid hydroperoxides. The present study has revealed that the oxidation of lipids and the dysregulation of the lipid metabolism coexisted in LDs in the kidney cells, which has provided a potential new target for diagnosis and new insights into CKD.

## 1. Introduction

Chronic kidney disease (CKD) is a prevalent non-infectious chronic disease throughout the world. Approximately 10% of the world’s population suffers from CKD, and more than 1.2 million people die from it every year [1,2,3]. CKD has been considered a global health issue, which both affects patients’ quality of life and brings a heavy economic burden to society. In addition, CKD has become an important risk factor for morbidity and mortality of cardiovascular diseases, leading to more severe and more complex health problems [2,4].

Until now, the pathological mechanisms that trigger CKD and its progression had not been completely elucidated [5]. Previous literature has revealed some potential factors that may be involved in CKD progression, such as oxidative stress, ectopic lipid accumulation, dyslipidemia, renal cell damage and dysfunction, and others [6,7,8,9]. Ectopic lipid accumulation refers to the abnormal state of lipid accumulation in LDs in non-adipose tissues (e.g., in the kidney) [9]. This disorder has been found to induce lipotoxicity by promoting inflammation, producing reactive oxygen species (ROS) and causing cell death, during which the LDs play a crucial role in oxidative stress and lipid dysregulation [10]. The LD, which serves as a universal storage organelle, is known as the center of lipid and energy homeostasis; moreover, it is a dynamic (or “mobile”) organelle in cells, with widely varied numbers, sizes, and compositions [11]. Such morphological diversity signifies the nutritional availability, the metabolic states, and the hormone levels [11,12,13]. Since the constituents in the LD include neutral lipids (e.g., triglycerides (TG) and cholesteryl esters (CE)) as the core and phospholipids (e.g., phosphatidylcholine (PC) and phosphatidylethanolamine (PE)) as the surface, the lipids in the LD have been recognized as a critical indicator responding to cellular physiology/pathology, metabolic function/disorders, and even the body health status. In terms of CKD, recent studies have revealed that LD disorder is associated with endoplasmic reticulum and mitochondria dysfunction and the variation of apolipoproteins [14,15]. However, while most studies have focused on LD-related genes and proteins, investigations targeting the LD lipids are quite limited. In addition, there is also a possibility that LD abnormalities in quality, not only in quantity, play a considerable role in the development of CKD.

Oxidative stress is one of the most vital factors in the development of CKD. The overproduced reactive oxygen species (ROS), as well as the inefficient antioxidant system, result in the imbalanced redox, which has been known to be associated with various pathological conditions and non-infectious chronic diseases, especially metabolic diseases, such as nonalcoholic fatty liver disease (NAFLD), type two diabetes, and CKD [16,17,18]. In cells, oxidative stress is commonly associated with damage to DNA, proteins, lipids, and other physiological molecules. For lipids, there are a variety of oxidation products, among which lipid hydroperoxides serve as a key intermediate of ROS-induced oxidative reactions [19]. Lipid peroxidation is also considered to cause ferroptosis via multiple pathways, such as by the modification of the membrane structure, an increase in membrane permeability, the rupture of the plasma membrane, and the dysregulation of the enzymatic processes [20,21]. Concerning CKD, recent studies have proved that such oxidation reactions negatively impact the structure and the function of the kidney, leading to autophagy and apoptosis of the cell and ischemia and fibrosis of the tissue [16]. However, whether it can and how oxidative stress influences organelles, especially LDs, has been rarely studied.

Although widely-used methods have been developed for evaluating lipid oxidation degrees (e.g., the malondialdehyde level), the direct detection of the oxidized lipid molecules (e.g., the lipid hydroperoxides) is preferable because it is a more intuitive and immediate approach to focus on the substances. Thanks to the development of the mass spectrometry (MS) approach, it is possible to quantify and (semi-)quantitate the lipid hydroperoxides from various biological samples [22,23]. In addition, nanoelectrospray ionization mass spectrometry (nanoESI-MS), combined with in-tip solvent microextraction, has enabled the in situ detection of the lipids from a single LD in living cells [24]. Therefore, it might be possible to investigate the oxidized lipid molecules in the LDs with regard to CKD, which has not yet been reported.

Hence, our current work focused on the changes in the LDs in human renal cells, specifically aiming to elucidate the chemical composition of LDs. The kidney tubular epithelial (HK-2) cells were supplemented with free fatty acids or oxidized lipoproteins in order to simulate the cellular-fat accumulation and the oxidative stress state of CKD. The LDs were evaluated by staining and nanoESI-MS. Based on the comprehensive lipidomic analysis of the LD lipids, we have revealed the lipid accumulation and the oxidation features, evaluated the usefulness of lipid hydroperoxides as a potential biomarker, and have investigated the LD morphological modulation-related lipid molecular alterations. Furthermore, the possible connection between lipid hydroperoxide accumulation and other lipid dysregulation in LDs was also discussed.

## 2. Materials and Methods

### 2.1. Chemicals

Dulbecco’s modified Eagle’s medium (DMEM, low-glucose), Dulbecco’s phosphate-buffered saline (DPBS), trypsin-EDTA, fetal bovine serum (FBS), penicillin, and streptomycin (100 U/mL) were purchased from Gibco (Carlsbad, CA, USA). Ammonium formate, trifluoroacetate, isopropanol, and methanol were purchased from Wako Pure Chemical Industries (Osaka, Japan). Palmitic acid (FA16:0), oleic acid (FA18:1), linoleic acid (FA18:2), linolenic acid (FA18:3), arachidonic acid (FA20:4), eicosapentaenoic acid (FA20:5), and docosahexaenoic acid (FA22:6) were purchased from Cayman Chemical Company (Ann Arbor, MI, USA). Unless otherwise specified, other reagents were purchased from Thermo Fisher Scientific Inc. (San Jose, CA, USA).

### 2.2. Preparation and Oxidation of Lipoprotein

EDTA plasma was collected from a healthy volunteer after fasting for 14 h. Plasma lipoproteins, including low-density lipoproteins (LDL) and high-density lipoproteins (HDL), were isolated and purified by ultracentrifugation (*d* = 1.019–1.225 kg/L for the upper fraction), high-performance size exclusion chromatography (Superose 6 column, GE Healthcare, Uppsala, Sweden), and centrifugal filtration (Ultracel 100 K for LDL and Ultracel 50 K for HDL; Merck Millipore Ltd., Carrigtwohill, Ireland), as described previously [25,26]. Next, the oxidized LDL (oxLDL) and oxidized HDL (oxHDL) were prepared by incubation of the native lipoprotein samples with CuSO_4_ in PBS (5 μM CuSO_4_ for 0.5 mg lipids as the final concentration) at 37 °C, as reported by Shrestha et al. [25], for 0.5 h, 1 h, 2 h, or 4 h. EDTA buffer was used throughout the isolation and purification processes of lipoproteins and the temperature was maintained at 4 °C to avoid auto-oxidation.

### 2.3. HK-2 Cell Culture and Treatment

HK-2 cells were obtained from American Type Culture Collection (Manassas, VA, USA). The cells were maintained in DMEM containing 10% FBS and 1% penicillin–streptomycin at 37 °C in a humidified incubator with 5% CO_2_. Before each experiment, cells were subcultured when the confluency reached 80−90%.

For the cell viability assay and oil red O staining, cells were seeded in 96-well plates (IWAKI Co., Ltd., Tokyo, Japan) at a concentration of 5 × 10^4^ cells/mL (i.e., 100 μL/well), while for the LD experiment, cells were seeded in 35 mm culture plates (IWAKI Co., Ltd.) at a concentration of 1 × 10^5^ cells/mL. The cells were maintained at 37 °C with 5% CO_2_ until a confluency of 80−90% was reached.

For LD inducement, HK-2 cells were supplemented with different fatty acids (see Section 2.1) or the prepared oxidized lipoproteins (see Section 2.2) into the culture medium (DMEM), then were incubated for 24 h. The grouped cells were annotated as “time-prepared oxLDL/oxHDL” according to the preparation time of the oxidized lipoproteins (e.g., 0.5 h-prepared oxLDL or 4 h-prepared oxHDL). The final concentration of each fatty acid was decided by its cytotoxicity. Specifically, for FA16:0, FA18:1, FA18:2, and FA18:3, the final concentrations added to medium were 100, 200, 300, 400, and 500 μM; for FA20:4, FA20:5, and FA22:6, the final concentrations were 50, 100, 150, 200, and 250 μM (*n* = 4 per treatment), while the final concentration of oxLDL and oxHDL was 0.2 mg/mL (*n* = 3 per treatment). The treated cells were seeded and incubated for 24 h prior to further experiments.

### 2.4. Cell Viability Test

Cell viability was determined using the CCK-8 assay (Dojindo Molecular Technologies, Rockville, MD, USA). After 24 h treatment, according to the manufacturer’s instructions, 10 μL of CCK-8 solution was added to each well. The cells were then incubated at 37 °C with 5% CO_2_ for 2 h. Finally, the absorbance was measured at 450 nm using a Wallac 1420 ARVOSx plate reader (PerkinElmer Co., Ltd., Waltham, MA, USA).

### 2.5. Oil Red O Staining

The cells treated in 96-well plates for 24 h were washed and rinsed with 1 × PBS and fixed in 10% formalin solution for 10 min. Next, the cells were washed twice with 200 μL PBS, incubated with 200 μL 60% isopropanol for 10 min, and were then removed. Oil red O dye (Wako Pure Chemical Industries), which is used for LD staining, was dissolved in isopropanol to prepare the stock solution (3 mg/mL). The working solution was prepared according to our previous study [27]; in brief, the stock solution (15 mL) was diluted with 10 mL of distilled water, and the final concentration was 1.8 mg/mL. The newly prepared working solution was thereafter filtrated using a 0.45 μm filter (Merck Millipore Ltd., Burlington, MA, USA) before use. The fixed cells were immersed in the oil red O working solution for 20 min and then washed with water to remove excess dye. Finally, the cells were observed under an IX71 microscope (Olympus, Tokyo, Japan), and the cell photos were taken using cellSens standard software 1.7.1 (Olympus). The achieved photographs were processed using ImageJ 1.53 k [28], and the LD amount and LD size were exported using the “Analyze Particles” function [29].

### 2.6. Glass Tip Preparation and LD Aspiration

A PC-10 pipette puller (Narishige Group, Tokyo, Japan) was used for preparing the borosilicate glass nanotips (length 55 mm, tip i.d. 2–6 μm, capillary i.d. 0.6 mm) according to the manufacturer’s instructions. The 1st step heating temperature was set at 60.5 °C, and the 2nd step heating temperature was set at 42−50 °C, depending on the tip diameter. The diameter of each prepared nanotip was confirmed under the microscope.

The LD sampling was conducted using an IM-11 three-dimensional mobile manipulator (Narishige Group), coupled with an IX71 inverted microscope in a bright field, as in our previous studies [24,27]. The culture medium of the cells was washed and then replaced with 0.6 mL 160 mM ammonium formate solution, and the LDs were aspirated into nanotips using the microinjector. The obtained single LD was immediately extracted with 10 µL of methanol/isopropanol (1:9 *v*/*v*, with 0.1% trifluoroacetate).

### 2.7. NanoESI-MS Analysis

NanoESI-MS analysis was carried out on an LTQ Orbitrap mass spectrometer (Thermo Fisher Scientific Inc.). The major parameters were set as follows: spray voltage in positive ionization mode, 2.0 kV; capillary temperature, 200 °C; resolution power, 60,000; max inject time, 100 ms; microscans, 1; MS/MS collision energy, 30 eV. The generated raw data (MS spectra) were processed using the workstation Xcalibur 2.2 (Thermo Fisher Scientific Inc.). The extracted ion current (XIC) mass tolerance was within 5.0 ppm, and the area under the XIC curve of each component was calculated and normalized as the percentage of the lipid class.

### 2.8. Statistics

All the data were expressed as mean ± standard deviation (SD). One-way ANOVA (with Tukey’s or Dunnett’s post hoc test) was calculated using GraphPad Prism 8.0 (GraphPad Software, La Jolla, CA, USA), of which the differences were considered significant at *p* < 0.05. Principal component analysis (PCA) was conducted using JMP pro 16.1 (SAS Institute Inc., Cary, NC, USA). The correlations between the variables were assessed using R 4.0 (www.project.org, accessed on 15 January 2022).

## 3. Results and Discussion

### 3.1. Lipid Accumulation Caused by Fatty Acid Treatment in HK-2 Cells

#### 3.1.1. Cell Viability

**Figure 1:**
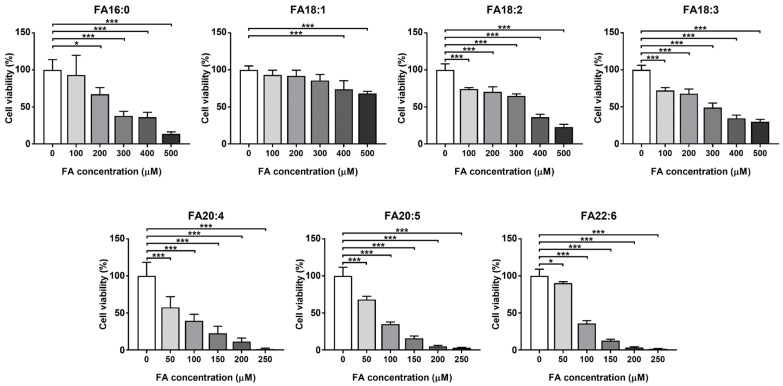
Cell viability of the fatty acid-treated cells, tested by CCK-8. * *p* < 0.05, *** *p* < 0.001, calculated by one-way ANOVA with the Dunnett’s post hoc test.

The HK-2 cell that was used in the current study is derived from a normal kidney and can retain functional characteristics in vitro, which has been widely used in order to study renal cellular physiology and lipid metabolism disorders [30,31,32,33]. The dietary common intake fatty acids, including FA16:0, FA18:1, FA18:2, FA18:3, FA20:4, FA20:5, and FA22:6, were investigated in order to induce lipid overloading HK-2 cells. In order to confirm the lipotoxicity of the excess free fatty acids in the cell, and to determine the appropriate dose for the lipid accumulation assays, we firstly tested the cytotoxicity of these fatty acids on the HK-2 cells. As a result, all of these fatty acids showed significant suppression of cell viability, even at the lowest concentration of each compound, and shared a similar decreasing trend in a dose-dependent manner (Figure 1). Among them, the saturated FA16:0, together with the C18 fatty acids FA18:1, FA18:2, and FA18:3, showed relatively lower cytotoxicity, all of which had IC50 values that were higher than 250 μM, while for the long-chain polyunsaturated fatty acids FA20:4, FA20:5, and FA22:6, much more severe cytotoxicity was observed.

The excess accumulation of free fatty acids is known to promote cellular dysfunction, to generate reactive oxygen species, to interfere with cell signaling pathways, and other damages [34]; especially in kidney cells, such accumulation has been proven to be a vital factor of renal lipotoxicity, which eventually leads to CKD and other lipid metabolism disorders [35]. It should be noted that C20 polyunsaturated fatty acids (PUFAs) are considered to attenuate lipotoxicity (typically induced by FA16:0) [36,37], while in our experiment, FA20:5 and FA22:6 exhibited even higher cytotoxicity than FA16:0. Based on the current data, for evaluating the lipid accumulation effects of fatty acid overloading, the concentration of FA16:0, FA18:1, FA18:2, and FA18:3 was selected as 200 μM, while the concentration of FA20:4, FA20:5, and FA22:6 was 100 μM. It should be mentioned that such high levels of fatty acids have been proven to be supraphysiological for the cells, resulting in abnormalities, such as the disruption of the lipid biosynthetic processes, the inducement of pyroptosis, the cause of metabolic inflammation, and others [38,39,40]. Nevertheless, the current experiment simulated the state of kidney cells under fatty acid overloading, and consequently expressed the supraphysiological changes.

#### 3.1.2. LD Morphology Dysregulated under Fatty Acid Overloading

**Figure 2:**
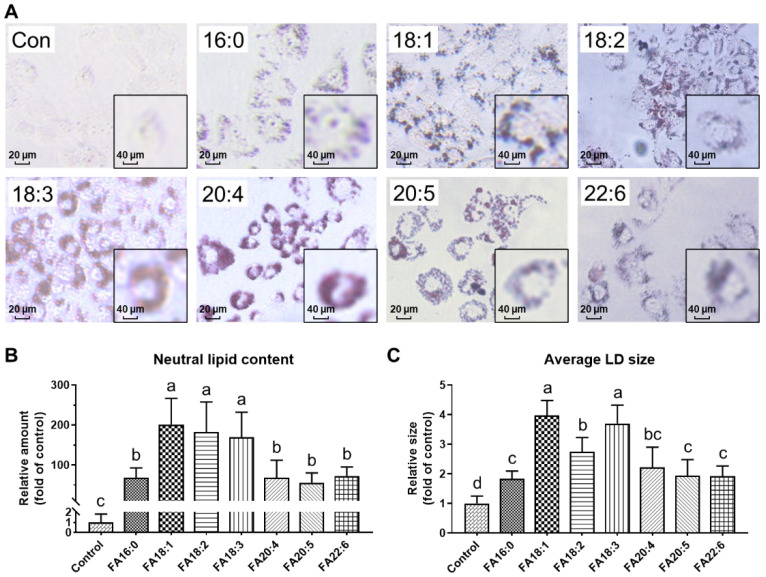
Neutral lipid accumulation in HK-2 cells treated with different fatty acids: 16:0, 18:0, 18:1, 18:2, 18:3, 20:4, 20:5, or 22:6. (**A**) Lipid droplet morphology, obtained by oil red O staining. (**B**) Area of the lipid droplets. (**C**) Size of the lipid droplets. a–d, bars with different letters differ at *p* < 0.05, calculated by one-way ANOVA with Tukey’s post hoc test.

We treated the HK-2 cells with different fatty acids of their appropriate concentration and conducted the oil red O staining in order to visualize the deposition of the neutral lipids. The representative photographs of the cells that were treated with different fatty acids after dyeing are shown in Figure 2A, in which the red particles indicate the deposited neutral lipids that are stored in LDs. There were almost no accumulated LDs observed in the control group, whereas in all of the other groups, a considerable number of LDs were detected, suggesting the confirmation of the neutral lipid accumulation in the LDs that were induced by fatty acid overloading. A subsequent calculation was performed in order to quantify these results; all of the investigated fatty acids caused a great increase in the neutral lipid content, accounting for 55.70−201.3 folds of control. Among them, the C18 fatty acids exhibited an even higher level (201.3 ± 65.3 folds for FA18:1, 182.2 ± 75.3 folds for FA18:2, and 169.6 ± 62.3 folds for FA18:3) than the long-chain PUFAs.

Studies have reported that PUFAs, especially at a low dose (e.g., 30 μM of FA22:6), exhibited protective effects against lipid accumulation and dysregulation [36], while our results have hinted at a new viewpoint that the C18 fatty acids might be more easily absorbed into LDs, which may cause a more significant growth of neutral lipid content and LD size. PUFAs tended not to be stored in the LDs but exert effects (both beneficial bioactivities and cytotoxicity) in the cytoplasmic matrix, which might explain why the cells were more sensitive to PUFAs.

#### 3.1.3. TG Profile in LDs Altered by Fatty Acid Intake

**Figure 3:**
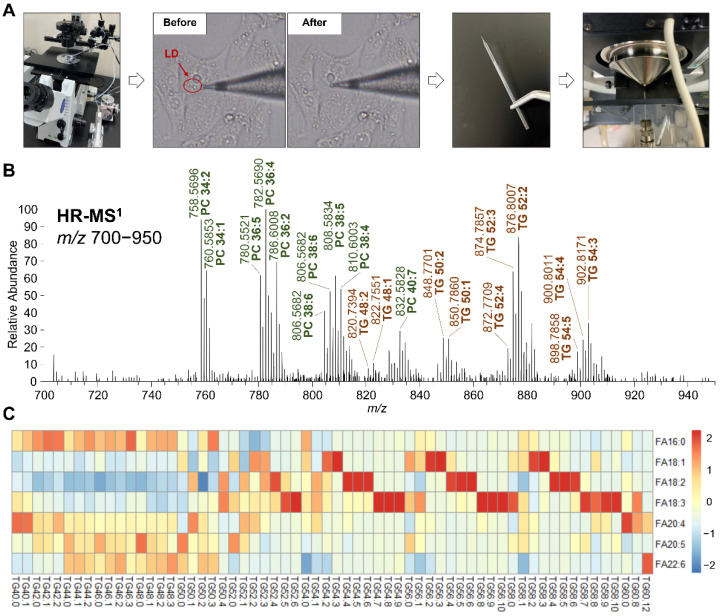
TG profile in LD analyzed using nanoESI−MS. (**A**) Workflow, including the control of glass capillary using 3D manipulator, aspiration of LD from cells, in-tip solvent microextraction, and sample spray into MS. (**B**) Representative high-resolution MS spectrum of LD, within the range from *m*/*z* 700 to 950. The representative PC and TG species are shown in green and red, respectively. (**C**) Heatmap of LD TG molecular species composition in the cells treated with different fatty acids.

In order to observe the influences on the lipids in the LDs from oxLDL or oxHDL, we performed LD aspiration and in-tip microextraction in order to isolate the LD lipids and detected them using nanoESI-MS (Figure 3A). While the oil red O staining was used for confirming the neutral lipid accumulation, nanoESI-MS-based lipidomics was subsequently used for clarifying the LD TG composition (i.e., molecular composition) and understanding how the lipids were accumulated. In total, 62 molecular species of TG have been identified using high-resolution MS (Figure 3B) in LDs that were induced by FA (Table S1). It is worth noting that this LD aspiration-nanoESI-MS technique could not completely exclude the other organelle fractions, even though we tried our best to aspirate only the LDs, which might be verified by western blotting using organelle-specific antibodies (e.g., mitochondria, endoplasmic reticulum, or lysosome). However, such a purity issue did not influence the MS results; the background interference signals from the matrix were considered to be negligible (Figure S1).

The relative abundance of TG species in the LDs depended on the type of FA that was supplemented to the cells (Figure 3C). When FA18:1 was supplemented, the TG species 54:2, 54:3, 56:2, 56:3, 58:2, and 58:3 were most predominant, which were mainly composed of oleoyl (18:1). In the case of FA18:2, the major TG species were 54:4, 54:5, 54:6, 56:4, 56:5, 56:6, 58:4, 58:5, and 58:6, which were abundant in linoleoyl (18:2), while the supplementation of FA18:3 resulted the increased contents of the linolenoyl (18:3)-rich TG, such as 54:7, 54:8, 54:9, 56:8, 56:9, 56:10, 58:7, 58:8, 58:9, and 58:10. These results showed that C18 fatty acids were likely to participate in TG formation and storage in the LDs and, consequently, they affect the composition of TG, i.e., they tend to construct the supplemented fatty acyl-contained TG species. However, when FA16:0, FA20:4, FA20:5, and FA22:6 were supplemented, there seemed to be no such changing behavior in the LD TG profile, suggesting that these fatty acids were not directly involved in the LD synthesis, though they induced the TG de novo biosynthesis and then influenced the size of the LDs. In addition, there were no oxidized lipids detected in FA-treated samples.

The treatment of FA to cells has profoundly affected the LD TG profile in our previous reports [24,27]. Currently, the results of FA18:1, FA18:2, and FA18:3 that were supplemented to HK-2 cells supported this viewpoint, as they were supposed to be taken up directly by the cells and be esterized as LD TG, while FA16:0, FA20:4, FA20:5, and FA22:6 showed no such function. These findings were consistent with our cytotoxicity and LD morphology results. To the best of our knowledge, this is the first study to directly analyze the profile and the fatty acyl composition of the lipids in LDs with regard to CKD. These results have indicated that the fatty acids that are supplemented to the cells might induce LDs to accumulate fats with different molecular compositions through multiple pathways in the body, such as by directly taking up the cells or by stimulating the biosynthesis of other fatty acids. Nevertheless, the detailed mechanisms and their regulating factors remain to be elucidated.

### 3.2. Alterations of LD Intact Lipids Induced by Oxidized Lipoprotein Treatment

As well as fat overloading, oxidative stress has also been recognized as a crucial factor in CKD [41,42]. Since the oxidized lipoproteins are known to express profound effects on the development of renal diseases [43,44], we have investigated the impact of oxLDL and oxHDL on the major intact lipids in LDs.

#### 3.2.1. Alterations of Neutral Lipids Induced by oxLDL

**Figure 4:**
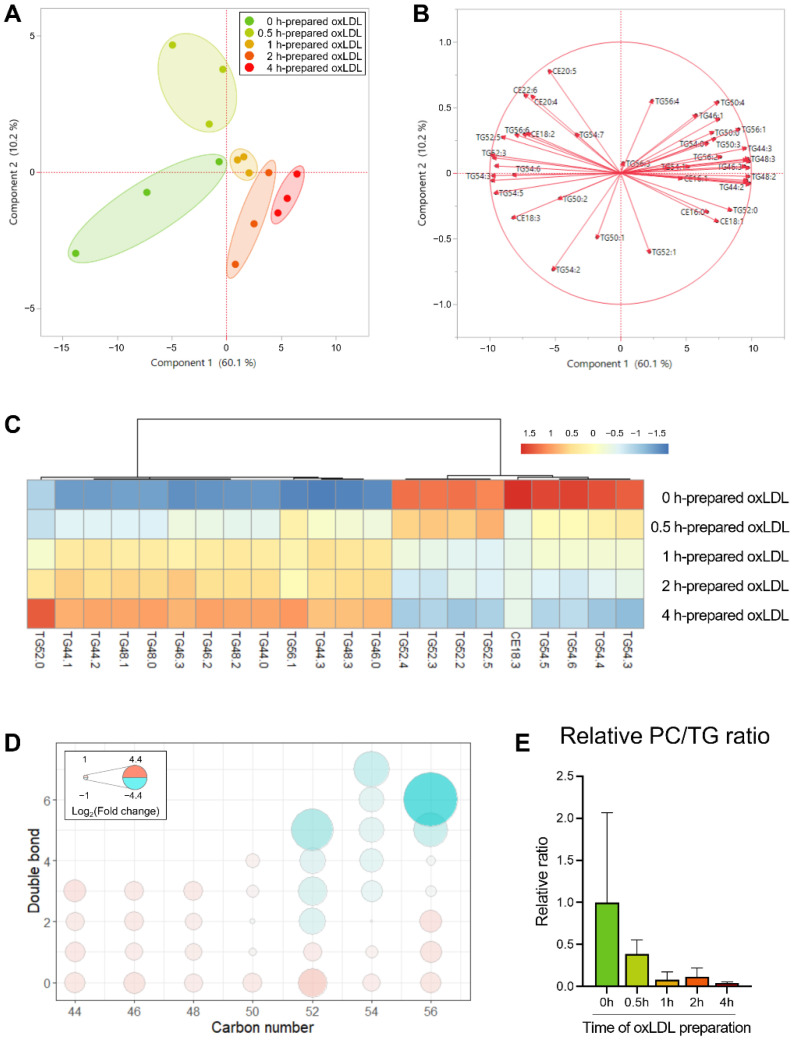
Score plot (**A**) and loading plot (**B**) of PCA revealed the distinguished profile of the intact neutral lipids in LDs from oxLDL-supplemented HK-2 cells. (**C**) Heatmap of the most significant varied LD neutral lipid species among different oxLDL-induced cells. (**D**) Bubble plots of LD TG profile changes in the cells supplemented using oxLDL with different preparation times. The color (ranging from red to cyan) and the bubble scales represent the degree of the difference, calculated as log_2_ (change fold of 2−4 h groups to 0−0.5 h groups). (**E**) PC/TG ratio of LDs in different groups.

In order to observe the influences on the lipids in LDs from oxLDL, we used the intact lipids (Table S2) as variables for performing PCA in order to visualize the lipidomic characteristic changes in the LDs. Here, the variables were divided into the LD core lipids (the neutral lipids, i.e., TG and CE) and the LD surface lipids (the phospholipids, i.e., PC and PE) in order to better discover the alterations. The neutral lipid profiles showed distinctive clusters in the scatter plot (Figure 4A), of which the first principal component, which accounted for 60.1% of the total variation, could explain most of the variations for these samples. In the loading plot (Figure 4B), most of the variables were distributed near the *x*-axis, positively or negatively. These results indicated that the first principal component should be the leading factor of the profile difference, suggesting that the neutral lipids, especially the TG species, can reflect the oxLDL-induced LD lipid alterations. On the other hand, the LD surface lipids and the phospholipid species did not show a pattern as obvious as the neutral lipids (Figure S2).

Consequently, we extracted the most significant lipid species based on their loading matrix ranking of the first principal component and compared their levels among the five groups (Figure 4C). Obviously, the 0 h and 0.5 h groups were similar, representing the non-oxidized/slight-oxidized state, and differed from the 2 h and 4 h groups (i.e., seriously oxidized). The supplementation of oxLDL with over 2 h of oxidation resulted in 13 TG being increased and 8 TG + 1 CE being decreased, suggesting that there might be a specific pattern of TG profile that is changing. Taking into consideration of the TG molecular composition, we noticed that all of the shorter carbon chain TG species (i.e., ≤50) were accumulated; moreover, in the C52−C56 TG species, the more unsaturated molecules (i.e., with more double bonds) decreased, typically TG52:5 and TG56:6, whereas the less unsaturated molecules increased, such as TG52:0 and TG56:2 (Figure 4D).

Similar results were found in the kidney tissue of the mice of the nonalcoholic steatohepatitis (NASH) model, in which the C58–C66 TG molecules with 12–18 double bonds were largely reduced [17]. In our present experiment, oxLDL caused the depletion of the long-chain polyunsaturated fatty acyls in the LDs, indicating the risk of steatosis, inflammation, dyslipidemia, and even carcinogenesis in the renal cells. Although similar findings were revealed in clinical and CKD-modelled animal studies [45,46], to the best of our knowledge, this is the first report on the distinguishment of the TG profile in the LDs from the renal cells with oxidative stress.

Furthermore, the LD morphology alteration also drew our attention, as these lipids regulate the LD surface properties. Specifically, the PC/TG ratio serves as an index of the LD size, which indicates a fat accumulation-induced LD abnormality [47]. As shown in Figure 4E, the PC/TG ratio exhibited a decreasing trend with the extended oxidation time, reaching 38.8 to 4.3% of the control (0 h) from 0.5 h to 4 h. Sufficient PC in the LDs contributes to the shielding the core lipids cytosol and preventing LD coalescence [48,49]; therefore, the lowered PC/TG ratio in the current study was considered to lead to the exposure of the hydrophobic LD core to the surrounding aqueous environment, as well as the inducement of fusion to reduce the LD surface-to-volume ratio, which caused the formation of the supersized LDs.

#### 3.2.2. Alterations of Phospholipids Induced by oxHDL

**Figure 5:**
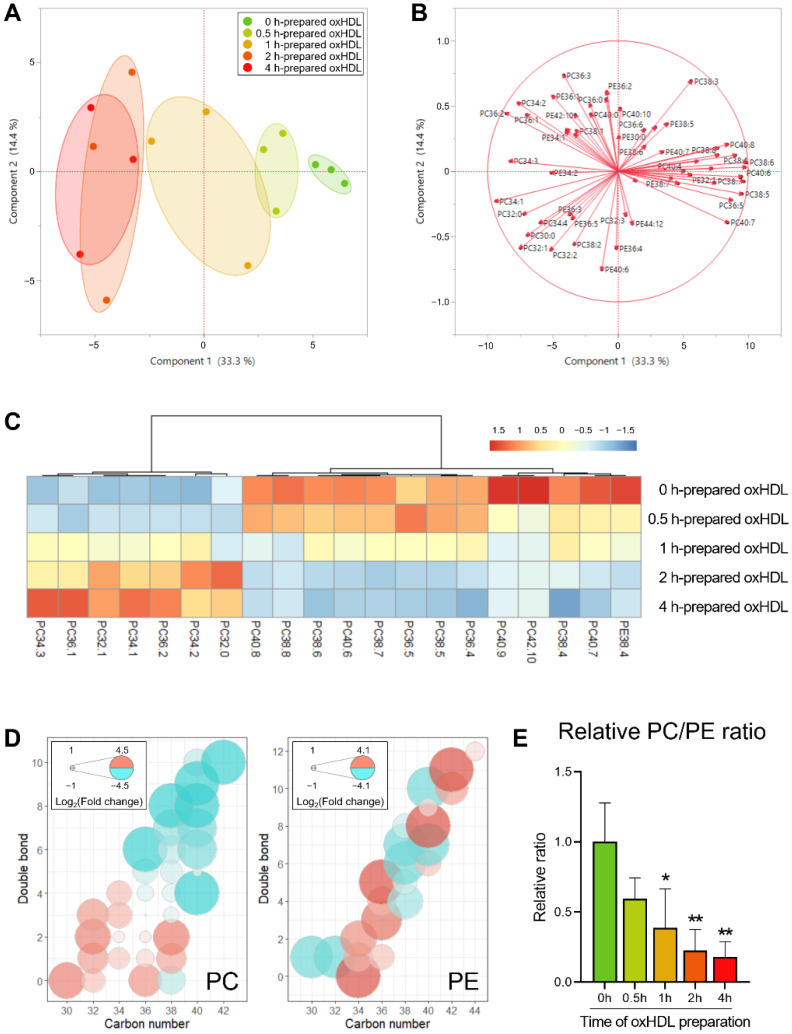
Score plot (**A**) and loading plot (**B**) of PCA revealed the distinguished profile of the intact neutral lipids in LDs from oxHDL-supplemented HK-2 cells. (**C**) Heatmap of the most significant varied LD phospholipid species among different oxHDL-induced cells. (**D**) Bubble plots of LD PC and LD PE profile changes in the cells supplemented using oxHDL with different preparation times. The color (ranging from red to cyan) and the bubble scales represent the degree of the difference, calculated as log_2_ (change fold of 2−4 h groups to 0−0.5 h groups). (**E**) PC/PE ratio of LDs in different groups, * *p* < 0.05, ** *p* < 0.01 vs. 0 h group, calculated by one-way ANOVA with Dunnett’s post hoc test.

The intact lipid profile was also investigated in the oxHDL-supplemented groups (Table S3), in which the phospholipid species in the scatter plot exhibited distinctive clusters (Figure 5A). Moreover, in the loading plot (Figure 5B), most of the phospholipid variables were distributed near the *x*-axis, positively or negatively. Consequently, we extracted the most significant lipid species based on their loading matrix ranking of the first principal component and compared their levels among the five groups (Figure 5C). In terms of the phospholipid molecular composition, the shorter and less unsaturated species were accumulated, typically the C32–C36 PC molecules with less than three double bonds, while the longer and more unsaturated phospholipids (e.g., PE38:4 and PC38:4) were lowered.

It is to be noted that the finding in the oxHDL-treated groups was similar to the groups treated with oxLDL, in which the fatty acyl chain length shortened and the unsaturation degree lowered. However, the more sensitive lipids in the oxHDL-treated groups were the phospholipids rather than the neutral lipids (Figure S3). Previous studies have shown PUFA depletion in CKD [50], while our present work suggested that the sources of the depleted PUFA might be variable, depending on the different oxidants. Nevertheless, the role, the oxidation mechanism, and the metabolic fate of both oxHDL and oxLDL need to be elucidated in the future. Moreover, concerning the molecular composition of PC and PE, it is worth noting that only PC showed the trend of PUFA depletion, but not PE (Figure 5D).

As the membrane composition of PC and PE played a critical role in regulating the LD morphology and the biophysical properties, and the PC/PE ratio was also investigated as a factor associated with packing defects [12] (Figure 5E). The PC/TG ratio exhibited a decreasing trend with the extended oxidation time, reaching 59.6 to 17.9% of the control (0 h) from 0.5 h to 4 h. The PC forms the cylindrical structures in the biological membranes, while the PE form the conical structures [38]. Therefore, a decreased PC/PE ratio is considered to promote packing defects and thereby favor the localization of perilipin to the LD surface, which contributes to lipid accumulation and LD growth [27]. According to the present results, the supplemented oxHDL decreased the PC/PE ratio, which suggested that it would aggravate the effect on the packing defects and result in LD enlargement. It should also be noted that the domain size characterization over a wide range of PC and PE molar proportions may be necessary in order to investigate the packing defects further, in order to prove the effects.

### 3.3. Lipid Hydroperoxidation Caused by Oxidized Lipoprotein Treatment in HK-2 Cells

**Figure 6:**
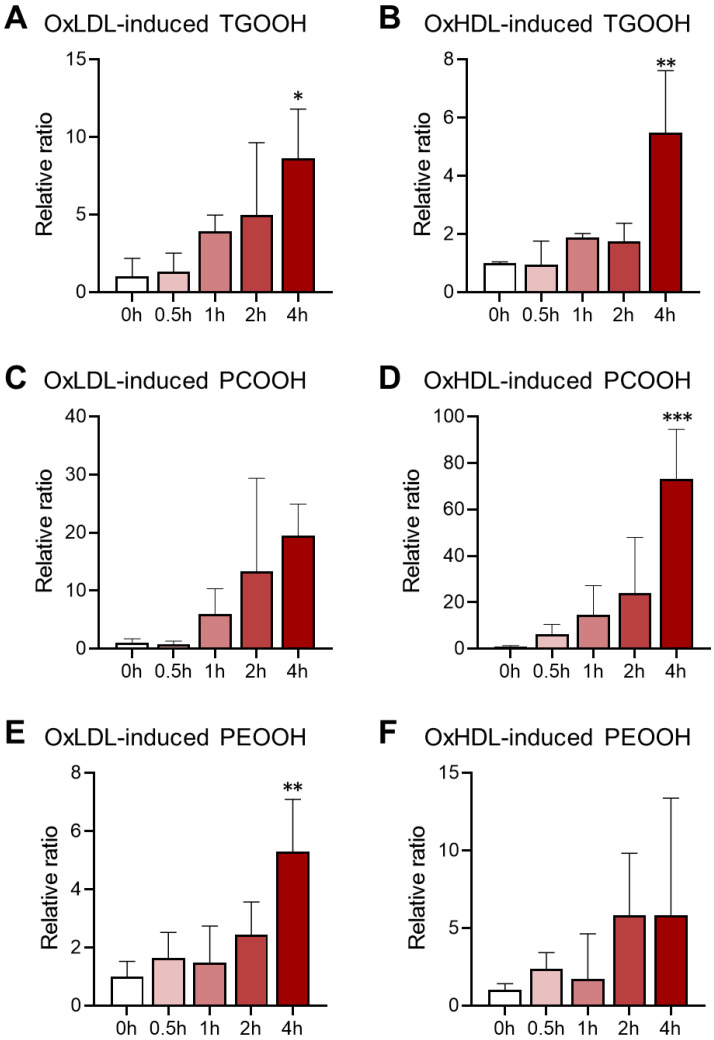
Accumulation of lipid hydroperoxides in LDs of oxidized lipoprotein-induced cells, including TGOOH (**A**,**B**), PCOOH (**C**,**D**), and PEOOH (**E**,**F**). * *p* < 0.05, ** *p* < 0.01, *** *p* < 0.001 vs. 0 h group, calculated by one-way ANOVA with Dunnett’s post hoc test.

In addition to the intact lipids, the lipid hydroperoxide (lipid-OOH) species were also detected in the LDs from the oxidized lipoprotein-treated HK-2 cells. The oxidized lipid molecules that were detected in our experiment included 30 species of TGOOH, 19 species of PCOOH, and 20 species of PEOOH in the oxLDL-treated groups, and 24 TGOOH, 26 PCOOH, and 12 PEOOH in the oxLDL-treated groups. The lipid hydroperoxidation level was calculated as the ratio of the total intact lipids to the total oxidized lipids, and the comparison, along with the lipoprotein oxidation time, is shown in Figure 6. Generally, all of the lipid hydroperoxide expressed a sharp increase after 4 h of oxidation, which was in a lipoprotein-oxidation-dependent manner. For the TGOOH, 4 h of oxidation for oxHDL and oxLDL led to 8.6 and 5.5 folds of the control group, respectively. In parallel, in the oxHDL-treated group, the PCOOH levels were much higher than in the oxLDL-treated group (e.g., 73.2-fold vs. 19.5-fold for 4 h oxidation). These results agreed with our finding that oxLDL was more effective than oxHDL in impacting the intact TG profile, while the oxHDL treatment resulted in an altered PC profile more severely than oxLDL. The PEOOH accumulated the least among these hydroperoxides in both the oxLDL- and the oxHDL-treated groups. While there has been a report on LD accumulation induced by oxidative stress in NAFLD [51], studies on the oxidative stress-related LD alteration in CKD are quite limited, particularly for the LD oxidation. Lipid hydroperoxides are known to connect with the vicious circle of abnormal lipid metabolism and energy production disturbance [17], yet, to the best of our knowledge, this is the first study to reveal lipid peroxidation in LDs from kidney cells.

CKD is associated not only with intact lipid profile alteration but also with lipid oxidation [8], while lipid hydroperoxides have been supposed to be the key intermediates in lipid oxidation [52]. Therefore, lipid hydroperoxidation in LDs suggested a signal of ectopic lipid accumulation combined with oxidative stress, which is, in our opinion, used for simulating the abnormal metabolic state of CKD. It should be noted that this is not yet a cell model of CKD, as there were more pathological characteristics and biological indexes to be verified. Another limitation is that the current method for LD lipid analysis focused on an individual (single LD) rather than a general view, which might also be a possible explanation for how the supplementation of oxLDL and oxHDL (especially for the longer preparation time) resulted in a considerable intragroup deviation. An effective alternative strategy is to isolate the total LD fraction from the cells using kits [53], but it is cumbersome and time consuming, which is risky for analyzing the susceptible oxidized lipids. Nevertheless, both oxLDL and oxHDL exhibited the potential to cause LD lipid peroxidation, which could therefore be proposed as prospective markers for indicating oxidative stress in CKD. In addition, the HK-2 cells that were used in our present work were considered more likely to be epithelial cells than the native proximal tubule cells, which was demonstrated by researchers using RNA-sequencing and proteomics. Since it is known that the native proximal tubule cells use fatty acids, but not glucose, as the energy source [54], it is necessary to elucidate the distinctive characteristics in the different kinds of renal cells in detail within subsequent works. In the future, this strategy will hopefully be applied to in vivo studies related to CKD (either animal models or clinical patients), as well as other metabolic diseases that are involved in oxidative stress and lipid dysregulation.

## 4. Conclusions

In conclusion, the current nanoESI-MS-based lipidomic study on lipid accumulation and oxidation in kidney cells in the CKD model proven that oxidative stress and lipid dysregulation played a crucial role in LD abnormality at the molecular level. Fat overloading resulted in characterized changes in the TG storage in the LDs, which depended on the kind of the supplemented fatty acids. In particular, PUFAs were putative to exert effects in the cytoplasmic matrix, rather than stored in the LDs. Moreover, the treatment of the oxidized lipoproteins caused both intact lipid profile alteration and oxidized lipid accumulation. The lipid hydroperoxides, especially TGOOH and PCOOH species, showed potential as diagnostic markers and evaluation indexes of LD oxidative stress. Our data have provided new insight into CKD development from the view of the LD lipid metabolism.

## Data Availability

Data are contained within the article or supplementary material.

## Supplementary Materials

The following supporting information can be downloaded at https://www.mdpi.com/article/10.3390/antiox11071387/s1: Figure S1: Comparison of the MS spectrum obtained from LD and cell matrix; Figure S2: Score plot (**A**) and loading plot (**B**) of PCA revealed the distinguished profile of the intact phospholipids in LDs from oxLDL-supplemented HK-2 cells; Figure S3: Score plot (**A**) and loading plot (**B**) of PCA revealed the distinguished profile of the intact neutral lipids in LDs from oxHDL-supplemented HK-2 cells; Table S1: TG composition in LDs of cells supplemented with different fatty acids; Table S2: Lipidomic profile in LDs of cells supplemented with oxLDL; Table S3: Lipidomic profile in LDs of cells supplemented with oxHDL.
