# Supplementary material for: Oxidative Stress and Lipid Dysregulation in Lipid Droplets: A Connection to Chronic Kidney Disease Revealed in Human Kidney Cells
Source: Antioxidants (Basel). 2022 Jul 18;11(7):1387. doi: 10.3390/antiox11071387 (PMC9312214; doi:10.3390/antiox11071387)
Supplement: Supplementary file 1 [file antioxidants-11-01387-s001.zip › antioxidants-1774341-supplementary.pdf]

Supplementary Materials

Table S1. TG composition in LDs of cells supplemented with different fatty acids

TG species	TG Composition (%)						
	FA16:0	FA18:1	FA18:2	FA18:3	FA20:4	FA20:5	FA22:6
TG40:0	0.26 ± 0.14	0.06 ± 0.07	0.10 ± 0.11	0.26 ± 0.23	0.41 ± 0.22	0.16 ± 0.15	0.08 ± 0.09
TG40:1	0.07 ± 0.03	0.00 ± 0.01	0.00 ± 0.00	0.04 ± 0.08	0.10 ± 0.12	0.02 ± 0.03	0.00 ± 0.00
TG42:0	1.41 ± 0.17	0.65 ± 0.44	0.33 ± 0.21	0.72 ± 0.47	1.15 ± 0.43	1.13 ± 0.61	0.66 ± 0.23
TG42:1	1.08 ± 0.15	0.21 ± 0.25	0.03 ± 0.06	0.31 ± 0.46	0.69 ± 0.32	0.66 ± 0.47	0.32 ± 0.21
TG42:2	0.07 ± 0.07	0.01 ± 0.01	0.00 ± 0.00	0.01 ± 0.02	0.06 ± 0.07	0.03 ± 0.03	0.01 ± 0.02
TG44:0	4.41 ± 0.14	2.69 ± 1.71	1.45 ± 0.31	2.33 ± 2.06	3.96 ± 0.62	5.16 ± 0.74	5.00 ± 0.60
TG44:1	4.83 ± 0.64	1.44 ± 1.53	0.66 ± 0.41	1.94 ± 1.08	3.74 ± 0.42	3.95 ± 0.93	4.78 ± 0.66
TG44:2	1.68 ± 0.69	0.43 ± 0.52	0.04 ± 0.05	0.27 ± 0.43	0.97 ± 0.40	1.13 ± 0.57	1.30 ± 0.49
TG46:0	7.79 ± 1.22	5.29 ± 2.56	2.76 ± 0.70	4.72 ± 2.59	5.96 ± 0.71	8.17 ± 1.28	6.61 ± 0.92
TG46:1	11.28 ± 0.53	3.89 ± 2.60	2.05 ± 0.64	4.77 ± 2.49	7.72 ± 0.79	9.42 ± 0.32	10.26 ± 1.09
TG46:2	6.49 ± 0.36	1.94 ± 1.91	1.08 ± 0.51	2.08 ± 2.06	4.21 ± 0.55	5.40 ± 0.25	6.83 ± 0.66
TG46:3	0.76 ± 0.35	0.20 ± 0.24	0.01 ± 0.01	0.25 ± 0.15	0.39 ± 0.23	0.41 ± 0.35	0.39 ± 0.27
TG48:0	5.45 ± 1.22	4.70 ± 1.94	3.49 ± 0.75	4.74 ± 3.11	5.31 ± 0.22	7.15 ± 0.53	5.81 ± 1.34
TG48:1	10.78 ± 0.41	4.91 ± 2.69	2.98 ± 0.60	3.94 ± 3.35	7.90 ± 0.61	9.69 ± 0.47	10.17 ± 0.49
TG48:2	9.88 ± 0.37	3.59 ± 2.77	2.27 ± 0.54	2.04 ± 1.86	6.57 ± 0.58	7.65 ± 0.56	9.25 ± 0.31
TG48:3	2.81 ± 0.34	0.89 ± 1.07	0.36 ± 0.26	0.64 ± 0.69	1.90 ± 0.66	1.40 ± 1.00	2.98 ± 0.83
TG50:0	0.77 ± 0.22	1.16 ± 1.03	0.40 ± 0.41	0.33 ± 0.55	0.44 ± 0.46	1.70 ± 0.76	1.35 ± 0.53
TG50:1	5.18 ± 0.29	3.56 ± 1.32	11.31 ± 6.40	6.65 ± 6.84	12.71 ± 3.80	7.91 ± 2.66	7.62 ± 2.26
TG50:2	6.41 ± 0.21	5.80 ± 0.98	4.09 ± 1.18	5.81 ± 2.95	6.46 ± 0.37	6.38 ± 0.63	6.95 ± 0.47
TG50:3	3.19 ± 0.18	1.29 ± 1.14	1.37 ± 0.58	2.04 ± 1.19	1.92 ± 0.65	2.17 ± 0.42	2.73 ± 0.68
TG50:4	0.16 ± 0.04	0.11 ± 0.14	0.83 ± 0.69	0.89 ± 0.62	0.10 ± 0.08	0.17 ± 0.15	0.18 ± 0.03
TG52:0	0.23 ± 0.06	0.48 ± 0.49	0.13 ± 0.13	0.47 ± 0.55	0.19 ± 0.23	0.58 ± 0.49	0.13 ± 0.12
TG52:1	1.25 ± 0.09	1.02 ± 0.98	3.46 ± 2.07	2.51 ± 2.48	3.48 ± 0.42	2.91 ± 0.20	1.88 ± 0.49
TG52:2	4.03 ± 0.66	10.60 ± 3.55	7.15 ± 3.41	8.40 ± 5.07	9.49 ± 2.22	6.59 ± 2.10	6.01 ± 1.25
TG52:3	2.30 ± 0.34	5.58 ± 1.65	5.54 ± 0.38	4.26 ± 1.70	3.90 ± 0.83	2.71 ± 1.13	3.04 ± 0.54
TG52:4	0.42 ± 0.15	0.33 ± 0.26	5.24 ± 1.84	2.86 ± 1.69	0.62 ± 0.24	0.26 ± 0.12	0.47 ± 0.28
TG52:5	0.02 ± 0.03	0.01 ± 0.01	0.84 ± 0.49	1.63 ± 1.58	0.07 ± 0.10	0.10 ± 0.12	0.00 ± 0.00
TG52:6	0.02 ± 0.03	0.03 ± 0.04	0.17 ± 0.14	2.11 ± 1.61	0.12 ± 0.10	0.11 ± 0.12	0.00 ± 0.00
TG54:0	1.16 ± 0.49	1.05 ± 0.87	0.75 ± 0.78	0.93 ± 0.81	0.75 ± 0.63	0.50 ± 0.38	0.16 ± 0.16
TG54:1	0.23 ± 0.13	0.28 ± 0.34	1.35 ± 0.97	1.32 ± 1.20	1.08 ± 0.38	0.39 ± 0.31	0.11 ± 0.09
TG54:2	0.56 ± 0.14	1.35 ± 1.02	1.00 ± 0.71	0.92 ± 0.56	0.69 ± 0.36	0.76 ± 0.55	0.48 ± 0.24
TG54:3	2.69 ± 0.17	27.72 ± 16.07	3.46 ± 0.58	3.13 ± 1.60	3.39 ± 0.67	2.74 ± 0.42	2.57 ± 0.51
TG54:4	0.90 ± 0.39	1.11 ± 0.47	5.65 ± 1.70	1.93 ± 1.45	1.60 ± 0.62	0.56 ± 0.13	0.85 ± 0.42
TG54:5	0.35 ± 0.13	0.51 ± 0.79	10.05 ± 3.75	2.33 ± 1.90	0.49 ± 0.39	0.23 ± 0.23	0.14 ± 0.13

TG species	TG Composition (%)						
	FA16:0	FA18:1	FA18:2	FA18:3	FA20:4	FA20:5	FA22:6
TG54:6	0.07 ± 0.02	0.06 ± 0.08	11.22 ± 5.29	3.03 ± 2.60	0.17 ± 0.15	0.28 ± 0.31	0.17 ± 0.24
TG54:7	0.00 ± 0.00	0.04 ± 0.07	0.05 ± 0.04	5.79 ± 5.06	0.00 ± 0.00	0.06 ± 0.07	0.00 ± 0.00
TG54:8	0.01 ± 0.02	0.00 ± 0.00	0.00 ± 0.00	3.21 ± 3.19	0.00 ± 0.00	0.00 ± 0.00	0.00 ± 0.01
TG54:9	0.28 ± 0.22	0.01 ± 0.01	0.03 ± 0.06	3.80 ± 3.03	0.02 ± 0.05	0.07 ± 0.08	0.37 ± 0.22
TG56:0	0.08 ± 0.07	0.27 ± 0.47	0.06 ± 0.12	0.24 ± 0.39	0.03 ± 0.04	0.23 ± 0.27	0.03 ± 0.05
TG56:1	0.20 ± 0.06	0.27 ± 0.25	0.03 ± 0.04	0.34 ± 0.39	0.16 ± 0.24	0.17 ± 0.24	0.02 ± 0.03
TG56:2	0.08 ± 0.06	0.24 ± 0.28	0.05 ± 0.09	0.04 ± 0.09	0.06 ± 0.02	0.03 ± 0.04	0.00 ± 0.00
TG56:3	0.08 ± 0.10	4.84 ± 3.05	0.18 ± 0.23	0.09 ± 0.15	0.05 ± 0.10	0.01 ± 0.02	0.01 ± 0.02
TG56:4	0.00 ± 0.00	0.31 ± 0.43	0.74 ± 0.75	0.10 ± 0.12	0.01 ± 0.02	0.00 ± 0.01	0.00 ± 0.00
TG56:5	0.00 ± 0.00	0.25 ± 0.41	2.38 ± 1.49	0.54 ± 0.68	0.09 ± 0.09	0.04 ± 0.05	0.12 ± 0.18
TG56:6	0.01 ± 0.02	0.00 ± 0.01	3.78 ± 1.70	0.47 ± 0.50	0.05 ± 0.06	0.26 ± 0.28	0.01 ± 0.02
TG56:8	0.00 ± 0.00	0.00 ± 0.00	0.03 ± 0.07	1.10 ± 1.04	0.17 ± 0.21	0.00 ± 0.00	0.00 ± 0.01
TG56:9	0.08 ± 0.12	0.01 ± 0.01	0.00 ± 0.00	1.27 ± 1.00	0.07 ± 0.07	0.03 ± 0.03	0.05 ± 0.08
TG56:10	0.00 ± 0.00	0.00 ± 0.00	0.00 ± 0.00	0.58 ± 0.58	0.00 ± 0.00	0.01 ± 0.02	0.00 ± 0.00
TG58:0	0.02 ± 0.03	0.08 ± 0.16	0.00 ± 0.00	0.16 ± 0.19	0.00 ± 0.00	0.06 ± 0.07	0.01 ± 0.01
TG58:1	0.07 ± 0.08	0.07 ± 0.15	0.00 ± 0.01	0.07 ± 0.09	0.07 ± 0.14	0.07 ± 0.11	0.00 ± 0.00
TG58:2	0.04 ± 0.05	0.11 ± 0.09	0.00 ± 0.00	0.00 ± 0.00	0.03 ± 0.04	0.02 ± 0.04	0.00 ± 0.00
TG58:3	0.01 ± 0.01	0.30 ± 0.24	0.03 ± 0.04	0.04 ± 0.08	0.00 ± 0.00	0.00 ± 0.00	0.00 ± 0.00
TG58:4	0.01 ± 0.02	0.03 ± 0.06	0.12 ± 0.17	0.00 ± 0.00	0.00 ± 0.00	0.00 ± 0.00	0.00 ± 0.00
TG58:5	0.02 ± 0.03	0.00 ± 0.01	0.37 ± 0.49	0.00 ± 0.00	0.00 ± 0.01	0.00 ± 0.00	0.00 ± 0.00
TG58:6	0.00 ± 0.00	0.00 ± 0.00	0.39 ± 0.35	0.12 ± 0.20	0.00 ± 0.00	0.09 ± 0.11	0.01 ± 0.02
TG58:7	0.00 ± 0.00	0.00 ± 0.00	0.05 ± 0.07	0.25 ± 0.47	0.01 ± 0.02	0.10 ± 0.11	0.00 ± 0.00
TG58:8	0.00 ± 0.00	0.15 ± 0.29	0.04 ± 0.04	0.28 ± 0.40	0.15 ± 0.18	0.00 ± 0.00	0.00 ± 0.01
TG58:9	0.00 ± 0.00	0.00 ± 0.00	0.00 ± 0.00	0.22 ± 0.33	0.08 ± 0.09	0.03 ± 0.03	0.00 ± 0.00
TG58:10	0.00 ± 0.00	0.05 ± 0.10	0.00 ± 0.00	0.55 ± 0.81	0.00 ± 0.00	0.11 ± 0.13	0.00 ± 0.00
TG60:0	0.04 ± 0.04	0.02 ± 0.03	0.04 ± 0.08	0.04 ± 0.08	0.10 ± 0.15	0.01 ± 0.01	0.03 ± 0.04
TG60:8	0.00 ± 0.00	0.00 ± 0.00	0.01 ± 0.02	0.09 ± 0.15	0.08 ± 0.01	0.01 ± 0.01	0.00 ± 0.00
TG60:12	0.00 ± 0.00	0.00 ± 0.00	0.00 ± 0.00	0.00 ± 0.00	0.02 ± 0.03	0.00 ± 0.00	0.03 ± 0.04

Table S2. Lipidomics profile in LDs of cells supplemented with oxLDL

Lipid species	Composition (%)				
	0 h	0.5 h	1 h	2 h	4 h
TG44:0	2.55% ± 1.40%	3.34% ± 0.23%	3.98% ± 0.29%	4.16% ± 0.41%	4.49% ± 0.15%
TG44:1	3.17% ± 1.38%	3.96% ± 0.25%	4.79% ± 0.24%	5.08% ± 0.42%	5.29% ± 0.27%
TG46:0	1.22% ± 0.87%	1.84% ± 0.12%	2.19% ± 0.19%	2.28% ± 0.30%	2.41% ± 0.13%
TG46:1	0.10% ± 0.14%	0.22% ± 0.07%	0.18% ± 0.17%	0.16% ± 0.08%	0.26% ± 0.02%
TG46:2	3.43% ± 1.66%	4.46% ± 0.38%	5.14% ± 0.20%	5.32% ± 0.45%	5.74% ± 0.18%
TG48:0	6.32% ± 1.67%	7.42% ± 0.60%	8.71% ± 0.42%	9.06% ± 0.65%	9.64% ± 0.30%
TG44:2	4.01% ± 1.10%	4.81% ± 0.32%	5.71% ± 0.27%	5.84% ± 0.49%	6.24% ± 0.45%
TG44:3	0.67% ± 0.65%	1.23% ± 0.08%	1.46% ± 0.13%	1.44% ± 0.08%	1.56% ± 0.15%
TG46:3	2.37% ± 1.29%	3.16% ± 0.29%	3.53% ± 0.16%	3.87% ± 0.10%	3.96% ± 0.17%
TG48:1	6.11% ± 1.20%	7.03% ± 0.45%	8.03% ± 0.30%	8.42% ± 0.54%	8.80% ± 0.23%
TG48:2	5.59% ± 1.07%	6.69% ± 0.51%	7.58% ± 0.21%	7.75% ± 0.46%	8.18% ± 0.35%
TG48:3	1.87% ± 0.77%	2.65% ± 0.19%	3.03% ± 0.05%	3.11% ± 0.19%	3.23% ± 0.21%
TG50:0	0.55% ± 0.41%	0.83% ± 0.24%	0.90% ± 0.20%	0.71% ± 0.62%	1.21% ± 0.07%
TG50:1	5.07% ± 0.31%	4.57% ± 0.09%	4.55% ± 0.22%	5.39% ± 0.86%	4.77% ± 0.22%
TG50:2	5.69% ± 0.80%	5.71% ± 0.11%	5.57% ± 0.17%	5.34% ± 0.65%	5.73% ± 0.17%
TG50:3	2.31% ± 0.36%	3.00% ± 0.07%	3.16% ± 0.12%	2.79% ± 0.41%	3.22% ± 0.24%
TG50:4	0.39% ± 0.25%	0.76% ± 0.02%	0.75% ± 0.08%	0.58% ± 0.07%	0.79% ± 0.10%
TG52:0	0.14% ± 0.14%	0.16% ± 0.09%	0.22% ± 0.06%	0.28% ± 0.06%	0.39% ± 0.08%
TG52:1	1.39% ± 0.50%	0.84% ± 0.44%	1.25% ± 0.08%	1.69% ± 0.93%	1.34% ± 0.19%
TG52:2	9.62% ± 4.83%	8.02% ± 2.06%	5.06% ± 1.08%	5.16% ± 1.14%	3.67% ± 0.78%
TG52:3	7.29% ± 3.42%	6.16% ± 1.40%	3.76% ± 0.49%	3.12% ± 0.14%	2.70% ± 0.46%
TG52:4	3.19% ± 0.74%	2.68% ± 0.39%	1.96% ± 0.10%	1.68% ± 0.22%	1.43% ± 0.17%
TG52:5	0.14% ± 0.06%	0.13% ± 0.08%	0.04% ± 0.02%	0.03% ± 0.03%	0.01% ± 0.01%
TG54:0	0.33% ± 0.21%	0.41% ± 0.09%	0.44% ± 0.06%	0.37% ± 0.19%	0.60% ± 0.14%
TG54:1	0.57% ± 0.38%	0.53% ± 0.10%	0.58% ± 0.06%	0.60% ± 0.21%	0.66% ± 0.07%
TG54:2	1.63% ± 0.49%	0.84% ± 0.09%	1.21% ± 0.04%	1.25% ± 0.51%	1.17% ± 0.19%
TG54:3	8.31% ± 1.94%	6.68% ± 0.17%	5.83% ± 0.30%	5.51% ± 0.17%	4.39% ± 0.65%
TG54:4	6.53% ± 1.60%	4.75% ± 0.18%	4.09% ± 0.23%	3.65% ± 0.15%	2.90% ± 0.52%
TG54:5	5.33% ± 1.41%	3.44% ± 0.12%	3.12% ± 0.06%	2.63% ± 0.42%	2.19% ± 0.20%
TG54:6	2.63% ± 0.20%	1.69% ± 0.09%	1.54% ± 0.07%	1.23% ± 0.17%	1.14% ± 0.11%
TG54:7	0.37% ± 0.24%	0.20% ± 0.05%	0.09% ± 0.03%	0.06% ± 0.04%	0.10% ± 0.08%
TG56:0	0.09% ± 0.09%	0.28% ± 0.09%	0.26% ± 0.04%	0.21% ± 0.14%	0.35% ± 0.05%
TG56:1	0.23% ± 0.22%	0.51% ± 0.06%	0.50% ± 0.03%	0.48% ± 0.13%	0.64% ± 0.06%
TG56:2	0.19% ± 0.17%	0.28% ± 0.16%	0.36% ± 0.05%	0.32% ± 0.12%	0.43% ± 0.03%
TG56:3	0.28% ± 0.11%	0.25% ± 0.24%	0.28% ± 0.03%	0.24% ± 0.08%	0.24% ± 0.04%
TG56:4	0.08% ± 0.07%	0.14% ± 0.06%	0.12% ± 0.02%	0.11% ± 0.07%	0.09% ± 0.03%
TG56:5	0.11% ± 0.11%	0.17% ± 0.11%	0.02% ± 0.01%	0.06% ± 0.05%	0.02% ± 0.02%
TG56:6	0.12% ± 0.13%	0.15% ± 0.15%	0.01% ± 0.01%	0.01% ± 0.02%	0.00% ± 0.00%

Lipid species	Composition (%)				
	0 h	0.5 h	1 h	2 h	4 h
PC30:0	0.45% ± 0.32%	0.10% ± 0.14%	0.14% ± 0.13%	0.31% ± 0.54%	0.14% ± 0.18%
PC32:0	1.22% ± 0.62%	0.87% ± 0.46%	0.89% ± 0.79%	2.11% ± 0.43%	2.34% ± 0.32%
PC32:1	1.91% ± 1.15%	1.17% ± 1.05%	4.56% ± 4.64%	4.46% ± 3.43%	3.37% ± 3.12%
PC32:2	0.13% ± 0.13%	0.04% ± 0.02%	0.01% ± 0.02%	0.09% ± 0.15%	0.00% ± 0.00%
PC32:3	0.04% ± 0.03%	0.01% ± 0.02%	0.00% ± 0.01%	0.00% ± 0.00%	0.03% ± 0.06%
PC34:0	0.02% ± 0.03%	0.02% ± 0.01%	0.01% ± 0.01%	0.04% ± 0.06%	0.06% ± 0.11%
PC34:1	18.97% ± 0.66%	13.41% ± 3.87%	24.42% ± 2.62%	30.87% ± 3.88%	47.88% ± 1.68%
PC34:2	21.87% ± 3.98%	21.50% ± 1.70%	19.41% ± 7.29%	16.01% ± 3.95%	12.84% ± 1.23%
PC34:3	0.41% ± 0.19%	0.32% ± 0.20%	0.17% ± 0.26%	0.38% ± 0.34%	0.16% ± 0.14%
PC34:4	0.30% ± 0.15%	0.07% ± 0.10%	0.11% ± 0.14%	0.25% ± 0.38%	0.13% ± 0.12%
PC34:5	0.00% ± 0.00%	0.00% ± 0.00%	0.00% ± 0.00%	0.14% ± 0.25%	0.00% ± 0.00%
PC36:0	0.01% ± 0.01%	0.01% ± 0.02%	0.01% ± 0.03%	0.01% ± 0.01%	0.09% ± 0.16%
PC36:1	3.14% ± 0.64%	3.29% ± 0.26%	2.58% ± 2.28%	5.52% ± 1.05%	8.15% ± 1.89%
PC36:2	14.22% ± 0.61%	13.72% ± 0.47%	13.99% ± 2.45%	13.47% ± 1.88%	11.19% ± 0.62%
PC36:3	5.33% ± 0.29%	6.05% ± 0.50%	4.39% ± 0.85%	3.31% ± 1.25%	1.00% ± 0.47%
PC36:4	10.83% ± 1.33%	11.40% ± 0.81%	8.70% ± 0.20%	7.80% ± 1.91%	5.34% ± 1.17%
PC36:5	3.14% ± 0.88%	4.71% ± 1.01%	2.00% ± 1.75%	3.24% ± 1.28%	0.79% ± 0.21%
PC38:0	0.00% ± 0.00%	0.00% ± 0.01%	1.09% ± 1.78%	0.19% ± 0.18%	0.04% ± 0.07%
PC38:1	0.06% ± 0.10%	0.01% ± 0.02%	0.06% ± 0.09%	0.25% ± 0.19%	0.00% ± 0.00%
PC38:2	0.22% ± 0.21%	0.03% ± 0.03%	3.77% ± 6.17%	1.00% ± 0.95%	0.16% ± 0.18%
PC38:3	1.75% ± 0.39%	2.15% ± 0.50%	2.02% ± 1.06%	0.80% ± 0.26%	0.87% ± 0.18%
PC38:4	5.55% ± 0.43%	6.62% ± 0.80%	3.78% ± 0.64%	3.65% ± 0.72%	2.25% ± 0.11%
PC38:5	3.89% ± 0.65%	5.19% ± 0.62%	4.21% ± 2.13%	3.40% ± 0.66%	1.49% ± 0.22%
PC38:6	3.23% ± 0.59%	4.60% ± 0.55%	1.13% ± 1.01%	0.94% ± 0.43%	0.74% ± 0.07%
PC38:7	0.93% ± 0.12%	1.39% ± 0.25%	0.32% ± 0.29%	0.27% ± 0.24%	0.19% ± 0.32%
PC38:8	0.00% ± 0.01%	0.00% ± 0.00%	0.00% ± 0.00%	0.00% ± 0.00%	0.00% ± 0.00%
PC40:0	0.02% ± 0.01%	0.00% ± 0.00%	1.39% ± 2.32%	0.00% ± 0.00%	0.02% ± 0.04%
PC40:4	0.05% ± 0.04%	0.03% ± 0.03%	0.01% ± 0.02%	0.00% ± 0.00%	0.00% ± 0.00%
PC40:5	0.03% ± 0.04%	0.00% ± 0.00%	0.00% ± 0.00%	0.58% ± 1.00%	0.06% ± 0.10%
PC40:6	1.28% ± 0.45%	1.79% ± 0.33%	0.50% ± 0.43%	0.67% ± 0.26%	0.41% ± 0.13%
PC40:7	0.68% ± 0.07%	1.04% ± 0.05%	0.27% ± 0.23%	0.26% ± 0.10%	0.25% ± 0.05%
PC40:8	0.15% ± 0.11%	0.18% ± 0.17%	0.03% ± 0.06%	0.00% ± 0.00%	0.00% ± 0.00%
PC40:9	0.20% ± 0.06%	0.27% ± 0.20%	0.03% ± 0.06%	0.00% ± 0.00%	0.00% ± 0.00%
PE30:0	0.07% ± 0.13%	0.00% ± 0.00%	0.00% ± 0.00%	0.00% ± 0.00%	0.00% ± 0.00%
PE30:1	0.28% ± 0.49%	24.70% ± 29.73%	4.87% ± 4.97%	3.11% ± 5.39%	9.69% ± 9.84%
PE32:0	0.00% ± 0.00%	0.33% ± 0.57%	0.13% ± 0.23%	0.00% ± 0.00%	0.00% ± 0.00%
PE32:1	1.15% ± 1.74%	0.65% ± 1.13%	0.00% ± 0.00%	0.00% ± 0.00%	0.00% ± 0.00%
PE32:2	0.00% ± 0.00%	0.00% ± 0.00%	3.65% ± 6.32%	5.13% ± 8.89%	3.93% ± 3.86%
PE32:3	0.00% ± 0.00%	0.24% ± 0.42%	0.11% ± 0.19%	0.00% ± 0.00%	0.00% ± 0.00%
PE34:0	1.30% ± 1.43%	0.78% ± 1.35%	0.00% ± 0.00%	11.22% ± 10.61%	0.00% ± 0.00%

Lipid species	Composition (%)				
	0 h	0.5 h	1 h	2 h	4 h
PE34:1	8.10% ± 5.27%	0.44% ± 0.47%	2.12% ± 2.01%	8.73% ± 9.80%	3.95% ± 3.96%
PE34:2	2.80% ± 1.18%	1.32% ± 1.30%	1.18% ± 1.21%	2.27% ± 1.14%	3.26% ± 5.64%
PE34:3	3.71% ± 6.43%	0.00% ± 0.00%	0.00% ± 0.00%	0.65% ± 1.13%	0.00% ± 0.00%
PE34:4	0.00% ± 0.00%	0.00% ± 0.00%	0.47% ± 0.81%	0.00% ± 0.00%	0.24% ± 0.41%
PE36:0	1.66% ± 2.36%	0.23% ± 0.40%	1.09% ± 0.98%	0.00% ± 0.00%	0.57% ± 0.99%
PE36:1	17.19% ± 6.68%	15.24% ± 10.91%	12.72% ± 10.67%	15.87% ± 10.70%	6.43% ± 6.28%
PE36:2	24.58% ± 13.61%	16.87% ± 14.25%	11.21% ± 7.87%	7.08% ± 12.26%	5.51% ± 8.89%
PE36:3	0.94% ± 0.86%	0.78% ± 1.35%	0.00% ± 0.00%	0.00% ± 0.00%	0.00% ± 0.00%
PE36:4	1.97% ± 1.77%	0.00% ± 0.00%	0.57% ± 0.98%	1.45% ± 2.51%	1.97% ± 3.41%
PE36:5	1.15% ± 1.23%	0.00% ± 0.00%	0.00% ± 0.00%	3.63% ± 6.28%	0.00% ± 0.00%
PE38:4	9.12% ± 5.30%	2.18% ± 2.66%	6.26% ± 5.60%	5.29% ± 5.87%	1.10% ± 1.90%
PE38:5	17.32% ± 9.98%	7.10% ± 5.66%	1.48% ± 1.80%	3.89% ± 6.74%	1.32% ± 2.29%
PE38:6	0.21% ± 0.37%	2.03% ± 1.77%	0.88% ± 1.22%	0.00% ± 0.00%	0.00% ± 0.00%
PE40:6	1.33% ± 1.53%	0.42% ± 0.73%	3.43% ± 4.57%	1.55% ± 2.69%	0.00% ± 0.00%
PE40:7	4.69% ± 8.12%	25.44% ± 6.63%	29.96% ± 15.12%	25.42% ± 21.36%	56.25% ± 18.73%
PE40:8	0.69% ± 1.20%	0.00% ± 0.00%	0.00% ± 0.00%	0.00% ± 0.00%	0.00% ± 0.00%
PE40:9	0.00% ± 0.00%	0.15% ± 0.26%	0.50% ± 0.87%	0.00% ± 0.00%	0.00% ± 0.00%
PE40:10	1.73% ± 3.00%	1.09% ± 1.11%	0.81% ± 0.80%	1.01% ± 1.75%	1.74% ± 3.01%
PE44:12	0.00% ± 0.00%	0.00% ± 0.00%	18.56% ± 29.18%	3.69% ± 3.63%	4.06% ± 7.02%
CE16:0	0.27% ± 0.23%	1.50% ± 0.10%	0.98% ± 1.37%	4.61% ± 1.98%	7.37% ± 1.53%
CE16:1	0.30% ± 0.30%	1.22% ± 0.17%	0.86% ± 0.86%	2.11% ± 2.10%	3.21% ± 2.92%
CE18:0	0.00% ± 0.00%	0.00% ± 0.00%	0.04% ± 0.03%	0.00% ± 0.00%	0.06% ± 0.10%
CE18:1	5.18% ± 1.21%	10.87% ± 1.55%	17.71% ± 4.07%	41.69% ± 5.26%	74.11% ± 11.05%
CE18:2	84.27% ± 4.30%	73.27% ± 0.60%	74.04% ± 4.32%	48.90% ± 0.95%	13.74% ± 6.57%
CE18:3	1.36% ± 0.55%	0.00% ± 0.00%	0.00% ± 0.00%	0.00% ± 0.00%	0.00% ± 0.00%
CE20:4	7.44% ± 3.34%	10.92% ± 0.59%	6.22% ± 0.84%	2.70% ± 2.36%	1.51% ± 1.89%
CE20:5	0.40% ± 0.14%	1.00% ± 0.39%	0.14% ± 0.12%	0.00% ± 0.00%	0.00% ± 0.00%
CE22:6	0.78% ± 0.26%	1.22% ± 0.42%	0.00% ± 0.01%	0.00% ± 0.00%	0.00% ± 0.00%

Table S3. Lipidomics profile in LDs of cells supplemented with oxHDL

Lipid species	Composition (%)				
	0 h	0 h	0 h	0 h	0 h
TG44:0	2.00% ± 1.03%	0.53% ± 0.68%	1.10% ± 1.22%	2.45% ± 1.39%	0.53% ± 0.87%
TG44:1	1.43% ± 0.67%	0.81% ± 0.71%	1.55% ± 0.86%	2.67% ± 1.85%	0.74% ± 1.28%
TG44:2	0.29% ± 0.14%	0.26% ± 0.40%	0.36% ± 0.59%	1.02% ± 1.05%	0.00% ± 0.00%
TG44:3	0.00% ± 0.00%	0.05% ± 0.09%	0.03% ± 0.05%	0.07% ± 0.13%	0.02% ± 0.04%
TG46:0	2.81% ± 1.35%	1.05% ± 1.01%	2.51% ± 1.86%	3.45% ± 1.79%	1.82% ± 1.66%
TG46:1	2.99% ± 1.44%	3.34% ± 1.47%	4.23% ± 2.24%	5.23% ± 3.12%	7.86% ± 8.02%
TG46:2	1.76% ± 0.96%	1.51% ± 0.83%	1.92% ± 1.04%	3.18% ± 2.18%	1.00% ± 1.21%
TG46:3	0.02% ± 0.02%	0.16% ± 0.25%	0.41% ± 0.70%	0.69% ± 0.78%	0.00% ± 0.00%
TG48:0	2.57% ± 1.08%	0.77% ± 0.81%	1.32% ± 0.92%	2.99% ± 0.81%	3.29% ± 1.44%
TG48:1	3.99% ± 1.25%	4.58% ± 1.21%	5.25% ± 1.84%	6.27% ± 2.04%	9.40% ± 1.61%
TG48:2	3.09% ± 0.98%	3.78% ± 1.70%	4.43% ± 2.09%	4.91% ± 2.73%	4.31% ± 1.73%
TG48:3	0.72% ± 0.23%	0.56% ± 0.62%	1.15% ± 1.52%	1.73% ± 1.31%	1.17% ± 1.47%
TG50:0	0.49% ± 0.36%	0.04% ± 0.04%	0.42% ± 0.72%	0.92% ± 0.41%	1.65% ± 2.72%
TG50:1	6.16% ± 0.67%	5.84% ± 0.73%	6.62% ± 1.72%	6.86% ± 2.30%	18.75% ± 2.27%
TG50:2	6.44% ± 0.11%	7.29% ± 0.34%	7.51% ± 1.63%	6.53% ± 0.89%	12.83% ± 3.02%
TG50:3	2.54% ± 0.30%	2.24% ± 0.50%	2.55% ± 0.59%	2.56% ± 0.68%	1.50% ± 0.21%
TG50:4	0.21% ± 0.14%	0.16% ± 0.18%	0.39% ± 0.36%	0.48% ± 0.40%	0.00% ± 0.00%
TG52:0	0.04% ± 0.03%	0.05% ± 0.08%	0.11% ± 0.12%	0.23% ± 0.12%	0.31% ± 0.53%
TG52:1	0.56% ± 0.49%	0.40% ± 0.30%	1.97% ± 0.77%	2.04% ± 0.71%	3.93% ± 2.95%
TG52:2	19.04% ± 3.81%	22.41% ± 2.57%	17.28% ± 8.84%	14.39% ± 10.12%	10.96% ± 10.28%
TG52:3	13.77% ± 3.39%	15.63% ± 2.61%	12.33% ± 6.22%	8.25% ± 4.75%	2.40% ± 1.31%
TG52:4	4.63% ± 0.99%	4.29% ± 0.61%	3.69% ± 1.15%	2.62% ± 0.67%	1.15% ± 0.53%
TG52:5	0.46% ± 0.26%	0.34% ± 0.25%	0.26% ± 0.13%	0.24% ± 0.26%	0.00% ± 0.00%
TG52:6	0.01% ± 0.02%	0.04% ± 0.07%	0.02% ± 0.03%	0.03% ± 0.05%	0.00% ± 0.00%
TG54:0	0.06% ± 0.03%	0.03% ± 0.03%	0.16% ± 0.11%	0.35% ± 0.25%	1.82% ± 0.89%
TG54:1	0.06% ± 0.04%	0.09% ± 0.10%	0.28% ± 0.33%	0.55% ± 0.16%	0.33% ± 0.57%
TG54:2	1.41% ± 0.19%	1.83% ± 1.05%	2.06% ± 0.06%	2.37% ± 1.41%	5.19% ± 3.44%
TG54:3	9.70% ± 0.39%	10.49% ± 1.66%	8.45% ± 1.48%	7.19% ± 1.58%	3.67% ± 2.73%
TG54:4	6.50% ± 0.42%	6.10% ± 0.77%	5.88% ± 1.34%	4.27% ± 1.09%	0.82% ± 0.48%
TG54:5	3.73% ± 0.33%	3.32% ± 1.63%	3.02% ± 1.65%	2.55% ± 1.37%	0.81% ± 0.85%
TG54:6	1.28% ± 0.17%	0.84% ± 0.52%	1.40% ± 1.08%	1.06% ± 0.85%	0.13% ± 0.22%
TG54:7	0.05% ± 0.07%	0.06% ± 0.08%	0.06% ± 0.11%	0.08% ± 0.10%	0.15% ± 0.26%
TG54:8	0.00% ± 0.00%	0.04% ± 0.03%	0.01% ± 0.02%	0.00% ± 0.00%	0.00% ± 0.00%
TG54:9	0.00% ± 0.00%	0.00% ± 0.00%	0.00% ± 0.00%	0.00% ± 0.00%	1.53% ± 0.73%
TG56:0	0.00% ± 0.00%	0.01% ± 0.02%	0.10% ± 0.18%	0.11% ± 0.10%	0.00% ± 0.00%
TG56:1	0.01% ± 0.01%	0.06% ± 0.06%	0.00% ± 0.00%	0.31% ± 0.27%	0.06% ± 0.11%
TG56:2	0.04% ± 0.06%	0.02% ± 0.03%	0.27% ± 0.18%	0.32% ± 0.07%	0.68% ± 0.71%
TG56:3	0.08% ± 0.10%	0.10% ± 0.18%	0.29% ± 0.26%	0.38% ± 0.05%	0.85% ± 0.74%

Lipid species	Composition (%)				
	0 h	0 h	0 h	0 h	0 h
TG56:4	0.05% ± 0.02%	0.17% ± 0.15%	0.12% ± 0.14%	0.16% ± 0.02%	0.00% ± 0.00%
TG56:5	0.29% ± 0.19%	0.24% ± 0.34%	0.09% ± 0.12%	0.15% ± 0.17%	0.04% ± 0.08%
TG56:6	0.62% ± 0.27%	0.25% ± 0.20%	0.05% ± 0.08%	0.12% ± 0.15%	0.00% ± 0.00%
TG56:8	0.04% ± 0.04%	0.02% ± 0.03%	0.00% ± 0.00%	0.01% ± 0.01%	0.06% ± 0.10%
TG56:9	0.00% ± 0.00%	0.00% ± 0.00%	0.00% ± 0.00%	0.00% ± 0.00%	0.03% ± 0.06%
TG56:10	0.00% ± 0.00%	0.00% ± 0.00%	0.03% ± 0.05%	0.00% ± 0.00%	0.00% ± 0.00%
TG58:0	0.00% ± 0.00%	0.02% ± 0.03%	0.01% ± 0.01%	0.01% ± 0.02%	0.00% ± 0.00%
TG58:1	0.00% ± 0.00%	0.02% ± 0.04%	0.06% ± 0.09%	0.11% ± 0.11%	0.00% ± 0.00%
TG58:2	0.02% ± 0.02%	0.02% ± 0.04%	0.08% ± 0.07%	0.09% ± 0.02%	0.07% ± 0.12%
TG58:3	0.00% ± 0.00%	0.00% ± 0.00%	0.06% ± 0.06%	0.01% ± 0.01%	0.11% ± 0.19%
TG58:4	0.00% ± 0.00%	0.00% ± 0.00%	0.03% ± 0.03%	0.00% ± 0.00%	0.00% ± 0.00%
TG58:5	0.02% ± 0.02%	0.11% ± 0.19%	0.02% ± 0.04%	0.00% ± 0.00%	0.00% ± 0.00%
TG58:6	0.00% ± 0.00%	0.02% ± 0.04%	0.02% ± 0.04%	0.00% ± 0.00%	0.00% ± 0.00%
TG58:7	0.00% ± 0.00%	0.00% ± 0.00%	0.00% ± 0.00%	0.01% ± 0.01%	0.00% ± 0.00%
TG58:8	0.00% ± 0.00%	0.01% ± 0.01%	0.00% ± 0.00%	0.00% ± 0.00%	0.00% ± 0.00%
PC30:0	0.02% ± 0.01%	0.08% ± 0.08%	0.14% ± 0.18%	0.50% ± 0.29%	0.28% ± 0.32%
PC32:0	0.47% ± 0.06%	0.37% ± 0.12%	0.45% ± 0.46%	1.17% ± 0.49%	0.93% ± 0.31%
PC32:1	0.43% ± 0.06%	0.82% ± 0.34%	1.50% ± 0.50%	2.82% ± 1.46%	2.85% ± 2.13%
PC32:2	0.02% ± 0.01%	0.04% ± 0.03%	0.05% ± 0.05%	0.31% ± 0.35%	0.16% ± 0.22%
PC32:3	0.00% ± 0.01%	0.00% ± 0.00%	0.08% ± 0.13%	0.00% ± 0.00%	0.02% ± 0.04%
PC34:1	10.28% ± 0.72%	11.98% ± 0.49%	16.21% ± 2.43%	21.36% ± 2.86%	26.22% ± 3.50%
PC34:2	16.77% ± 0.89%	17.81% ± 0.90%	20.52% ± 2.56%	22.83% ± 2.67%	21.30% ± 3.43%
PC34:3	0.37% ± 0.10%	0.58% ± 0.23%	1.00% ± 0.03%	1.12% ± 0.10%	1.79% ± 0.34%
PC34:4	0.06% ± 0.03%	0.19% ± 0.10%	0.15% ± 0.13%	0.42% ± 0.27%	0.25% ± 0.21%
PC36:0	0.00% ± 0.00%	0.00% ± 0.00%	0.00% ± 0.00%	0.01% ± 0.02%	0.00% ± 0.01%
PC36:1	2.01% ± 0.20%	1.85% ± 0.37%	2.62% ± 0.70%	2.83% ± 0.98%	3.70% ± 0.73%
PC36:2	11.02% ± 0.38%	11.69% ± 0.18%	12.99% ± 1.90%	14.19% ± 1.85%	15.09% ± 1.29%
PC36:3	6.63% ± 0.22%	6.30% ± 0.44%	6.77% ± 1.05%	7.26% ± 0.54%	6.70% ± 0.50%
PC36:4	13.56% ± 0.33%	13.40% ± 0.43%	11.45% ± 1.25%	9.06% ± 1.61%	7.83% ± 0.78%
PC36:5	5.56% ± 0.38%	6.81% ± 0.49%	4.24% ± 2.32%	2.50% ± 0.29%	2.32% ± 1.15%
PC36:6	0.01% ± 0.02%	0.05% ± 0.06%	0.06% ± 0.09%	0.00% ± 0.00%	0.00% ± 0.00%
PC38:0	0.00% ± 0.00%	0.03% ± 0.04%	0.00% ± 0.00%	0.00% ± 0.01%	0.00% ± 0.01%
PC38:1	0.01% ± 0.01%	0.00% ± 0.00%	0.00% ± 0.00%	0.01% ± 0.02%	0.02% ± 0.04%
PC38:2	0.08% ± 0.10%	0.07% ± 0.08%	0.20% ± 0.18%	1.05% ± 1.67%	0.18% ± 0.20%
PC38:3	2.30% ± 0.17%	1.76% ± 0.09%	1.93% ± 0.40%	1.26% ± 0.96%	1.47% ± 0.33%
PC38:4	7.52% ± 0.06%	6.26% ± 0.11%	6.10% ± 0.21%	4.79% ± 1.10%	3.45% ± 0.82%
PC38:5	6.36% ± 0.23%	6.43% ± 0.50%	4.67% ± 1.52%	2.98% ± 0.50%	2.75% ± 0.64%
PC38:6	6.64% ± 0.08%	5.58% ± 0.21%	4.25% ± 0.94%	2.11% ± 0.88%	1.58% ± 0.34%
PC38:7	3.22% ± 0.30%	2.73% ± 0.15%	1.71% ± 1.29%	0.36% ± 0.32%	0.46% ± 0.35%
PC38:8	0.08% ± 0.03%	0.06% ± 0.06%	0.00% ± 0.00%	0.00% ± 0.00%	0.00% ± 0.00%

Lipid species	Composition (%)				
	0 h	0 h	0 h	0 h	0 h
PC40:0	0.00% ± 0.00%	0.00% ± 0.00%	0.00% ± 0.00%	0.00% ± 0.00%	0.00% ± 0.00%
PC40:10	0.00% ± 0.00%	0.07% ± 0.06%	0.12% ± 0.11%	0.02% ± 0.04%	0.00% ± 0.00%
PC40:4	0.08% ± 0.08%	0.00% ± 0.00%	0.00% ± 0.00%	0.00% ± 0.00%	0.00% ± 0.00%
PC40:5	0.00% ± 0.00%	1.12% ± 0.97%	0.85% ± 0.78%	0.51% ± 0.53%	0.45% ± 0.11%
PC40:6	2.19% ± 0.37%	1.79% ± 0.18%	0.94% ± 0.51%	0.21% ± 0.20%	0.17% ± 0.29%
PC40:7	2.23% ± 0.32%	1.13% ± 0.68%	0.86% ± 1.18%	0.29% ± 0.47%	0.00% ± 0.01%
PC40:8	0.81% ± 0.25%	0.72% ± 0.33%	0.16% ± 0.19%	0.03% ± 0.06%	0.03% ± 0.06%
PC40:9	1.22% ± 0.27%	0.28% ± 0.48%	0.00% ± 0.00%	0.00% ± 0.00%	0.00% ± 0.00%
PC42:10	0.03% ± 0.00%	0.00% ± 0.00%	0.00% ± 0.00%	0.00% ± 0.00%	0.00% ± 0.00%
PE30:0	0.00% ± 0.00%	0.00% ± 0.00%	4.02% ± 6.96%	0.00% ± 0.00%	0.00% ± 0.00%
PE30:1	3.08% ± 5.34%	8.11% ± 7.43%	0.00% ± 0.00%	0.00% ± 0.00%	0.00% ± 0.00%
PE32:1	1.61% ± 1.44%	0.00% ± 0.00%	0.00% ± 0.00%	0.00% ± 0.00%	0.00% ± 0.00%
PE34:0	0.00% ± 0.00%	0.00% ± 0.00%	0.00% ± 0.00%	2.69% ± 2.60%	0.00% ± 0.00%
PE34:1	0.91% ± 1.58%	3.34% ± 3.29%	4.50% ± 7.79%	8.58% ± 8.74%	5.62% ± 9.20%
PE34:2	1.15% ± 2.00%	1.34% ± 2.32%	2.31% ± 3.36%	2.26% ± 2.20%	13.43% ± 12.32%
PE36:1	8.72% ± 4.13%	10.56% ± 13.49%	10.84% ± 18.77%	26.46% ± 13.57%	20.57% ± 18.00%
PE36:2	21.84% ± 12.49%	27.79% ± 3.97%	25.61% ± 24.78%	22.46% ± 10.08%	27.11% ± 7.34%
PE36:3	0.60% ± 1.03%	0.49% ± 0.85%	0.35% ± 0.61%	0.44% ± 0.76%	10.77% ± 15.16%
PE36:4	1.73% ± 1.66%	1.45% ± 1.35%	16.18% ± 26.55%	7.47% ± 5.60%	2.63% ± 4.56%
PE36:5	0.00% ± 0.00%	0.14% ± 0.24%	0.00% ± 0.00%	0.00% ± 0.00%	2.37% ± 4.11%
PE38:4	32.81% ± 11.09%	15.77% ± 7.96%	10.41% ± 9.01%	3.63% ± 3.38%	4.84% ± 5.32%
PE38:5	15.81% ± 14.58%	14.51% ± 6.41%	6.87% ± 8.12%	2.17% ± 3.06%	11.15% ± 9.56%
PE38:6	0.00% ± 0.00%	3.60% ± 6.24%	0.00% ± 0.00%	0.00% ± 0.00%	0.29% ± 0.50%
PE38:7	1.07% ± 1.85%	0.00% ± 0.00%	0.00% ± 0.00%	0.00% ± 0.00%	0.00% ± 0.00%
PE38:8	0.97% ± 1.68%	0.70% ± 1.21%	0.02% ± 0.04%	0.70% ± 1.22%	0.00% ± 0.00%
PE40:10	7.16% ± 5.21%	2.55% ± 3.75%	0.00% ± 0.00%	0.00% ± 0.00%	0.00% ± 0.00%
PE40:6	2.01% ± 3.49%	6.85% ± 11.86%	8.74% ± 15.14%	21.72% ± 35.47%	0.00% ± 0.00%
PE40:7	0.53% ± 0.92%	1.81% ± 3.14%	0.00% ± 0.00%	0.00% ± 0.00%	0.00% ± 0.00%
PE40:8	0.00% ± 0.00%	0.00% ± 0.00%	0.00% ± 0.00%	0.00% ± 0.00%	0.31% ± 0.54%
PE40:9	0.00% ± 0.00%	0.63% ± 1.10%	0.26% ± 0.45%	0.98% ± 1.10%	0.00% ± 0.00%
PE42:10	0.00% ± 0.00%	0.20% ± 0.34%	0.00% ± 0.00%	0.21% ± 0.37%	0.66% ± 1.14%
PE42:11	0.00% ± 0.00%	0.00% ± 0.00%	0.00% ± 0.00%	0.24% ± 0.42%	0.00% ± 0.00%
PE44:12	0.00% ± 0.00%	0.15% ± 0.26%	9.90% ± 14.83%	0.00% ± 0.00%	0.24% ± 0.41%
CE16:0	1.22% ± 0.09%	0.31% ± 0.41%	3.75% ± 6.49%	0.60% ± 0.62%	0.00% ± 0.00%
CE16:1	0.86% ± 0.37%	0.51% ± 0.41%	3.23% ± 5.59%	0.63% ± 0.61%	0.00% ± 0.00%
CE18:0	0.01% ± 0.02%	0.00% ± 0.00%	0.00% ± 0.00%	0.00% ± 0.00%	0.00% ± 0.00%
CE18:1	9.53% ± 1.18%	5.54% ± 2.46%	4.12% ± 4.22%	10.89% ± 2.50%	6.56% ± 7.61%
CE18:2	71.67% ± 1.20%	82.22% ± 3.61%	56.45% ± 49.34%	82.38% ± 9.19%	91.93% ± 8.40%
CE18:3	2.03% ± 0.58%	1.83% ± 1.37%	7.10% ± 8.24%	0.85% ± 0.90%	0.00% ± 0.00%
CE20:4	12.03% ± 0.48%	8.18% ± 2.49%	19.10% ± 22.97%	4.03% ± 4.05%	1.04% ± 0.95%

Lipid species	Composition (%)				
	0 h	0 h	0 h	0 h	0 h
CE20:5	1.29% ± 0.42%	0.61% ± 0.62%	2.73% ± 4.04%	0.21% ± 0.37%	0.00% ± 0.00%
CE22:6	1.37% ± 0.59%	0.80% ± 0.59%	3.53% ± 5.37%	0.41% ± 0.50%	0.47% ± 0.82%

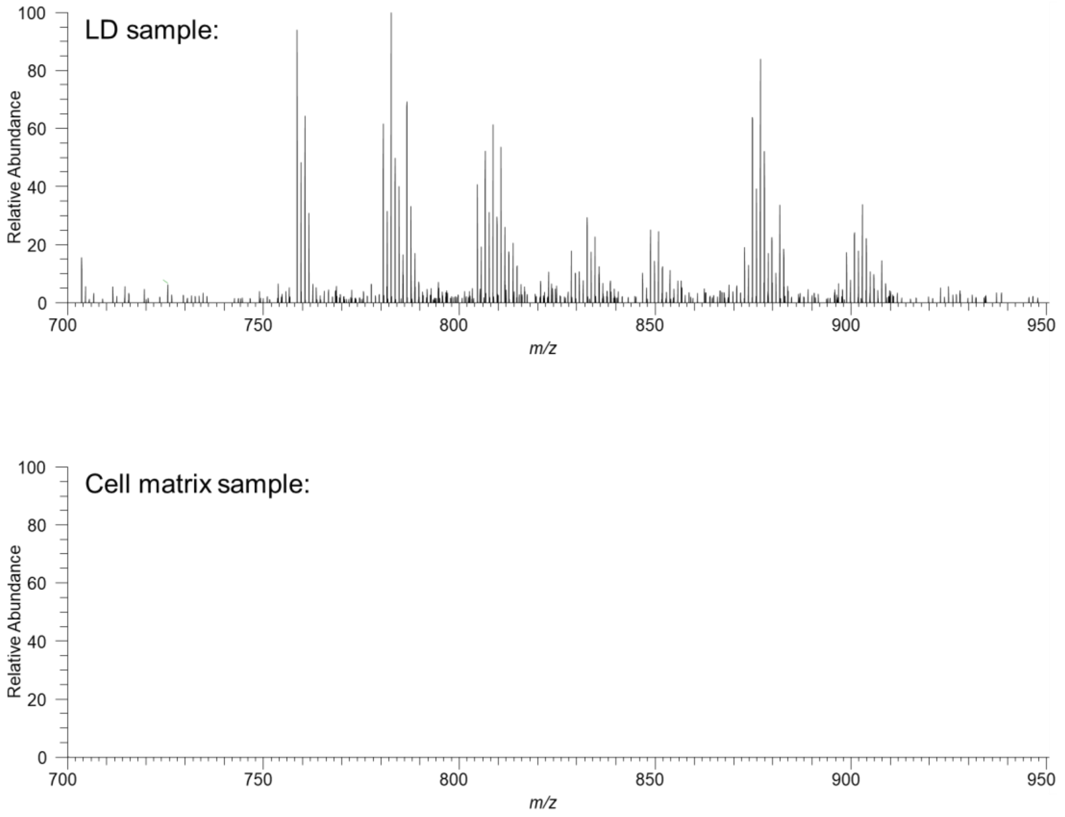

Figure S1: Comparison of the MS spectrum obtained from LD and from cell matrix.

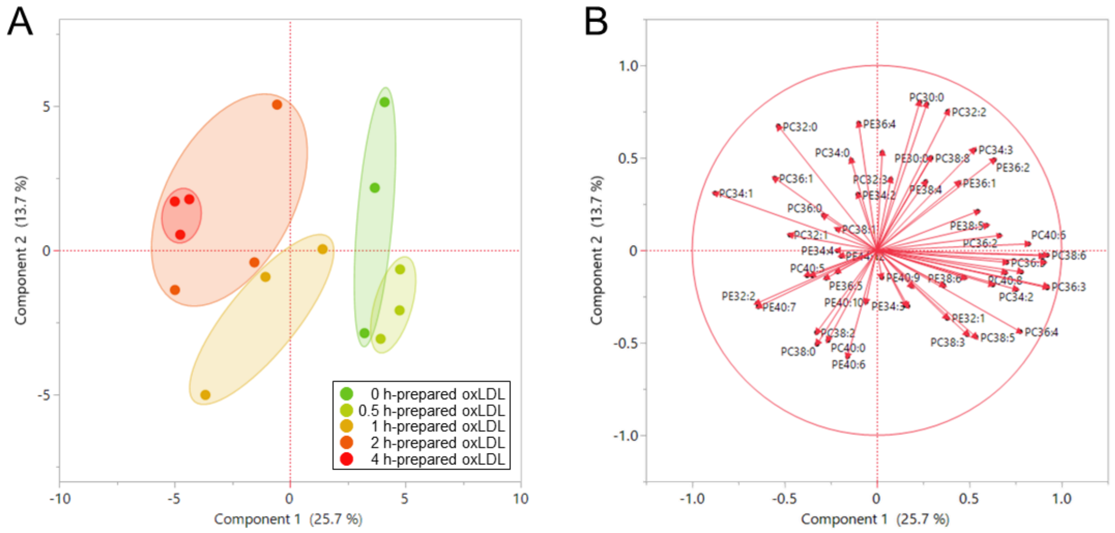

Figure S2. Score plot (**A**) and loading plot (**B**) of PCA revealed the distinguished profile of the intact phospholipids in LDs from oxLDL-supplemented HK-2 cells.

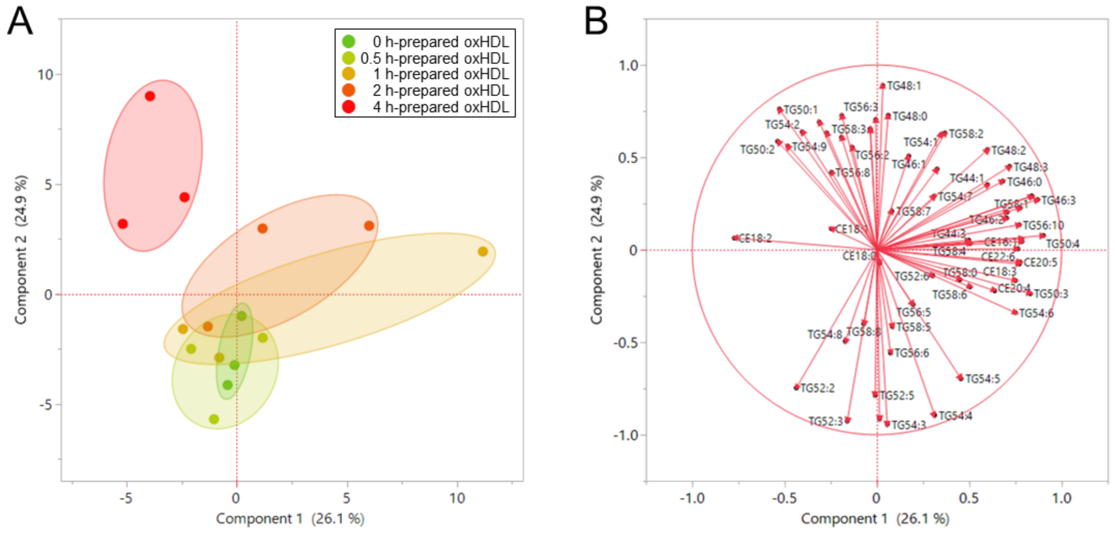

Figure S3. Score plot (**A**) and loading plot (**B**) of PCA revealed the distinguished profile of the intact neutral lipids in LDs from oxHDL-supplemented HK-2 cells.
